# Ancestral Genomes: a resource for reconstructed ancestral genes and genomes across the tree of life

**DOI:** 10.1093/nar/gky1009

**Published:** 2018-10-29

**Authors:** Xiaosong Huang, Laurent-Philippe Albou, Tremayne Mushayahama, Anushya Muruganujan, Haiming Tang, Paul D Thomas

**Affiliations:** 1 School of Life Sciences, Guangzhou University, Guangzhou 510006, China; 2 Division of Bioinformatics, Department of Preventive Medicine, Keck School of Medicine of USC, University of Southern California, Los Angeles, CA 90033, USA

## Abstract

A growing number of whole genome sequencing projects, in combination with development of phylogenetic methods for reconstructing gene evolution, have provided us with a window into genomes that existed millions, and even billions, of years ago. Ancestral Genomes (http://ancestralgenomes.org) is a resource for comprehensive reconstructions of these ‘fossil genomes’. Comprehensive sets of protein-coding genes have been reconstructed for 78 genomes of now-extinct species that were the common ancestors of extant species from across the tree of life. The reconstructed genes are based on the extensive library of over 15 000 gene family trees from the PANTHER database, and are updated on a yearly basis. For each ancestral gene, we assign a stable identifier, and provide additional information designed to facilitate analysis: an inferred name, a reconstructed protein sequence, a set of inferred Gene Ontology (GO) annotations, and a ‘proxy gene’ for each ancestral gene, defined as the least-diverged descendant of the ancestral gene in a given extant genome. On the Ancestral Genomes website, users can browse the Ancestral Genomes by selecting nodes in a species tree, and can compare an extant genome with any of its reconstructed ancestors to understand how the genome evolved.

## INTRODUCTION

The availability of completely sequenced genomes from organisms across the tree of life provides us with unprecedented opportunities to reconstruct the deep evolutionary past. Protein sequences can remain highly conserved over extended periods of time, indicating the effects of negative selection in removing deleterious mutations from populations. Clusters of orthologous groups—genes from different organisms that are inferred to derive from a single gene in the common ancestor of those organisms—have been used to infer the gene content of ancient ancestral genomes, such as that of the last universal common ancestor (1) (∼4 billion years ago) and the last eukaryotic common ancestor (2) (∼1.8 billion years ago). More recently, the development of tree reconciliation methods opens the door to ancestral genome reconstruction at any branch point in the tree of life (3,4).

Reconciled trees combine the information in the gene tree, usually obtained from protein sequences of related genes in different organisms, with prior knowledge of the species tree that relates those organisms. Because of this property, each node in a reconciled gene tree can be labeled with the evolutionary ‘event’ type that separated related genes: speciation, gene duplication, and horizontal gene transfer. Each speciation event can be labeled by the taxonomy designation of that group, such as Eukaryota (eukaryotes), or Metazoa (animals). A speciation event also represents a gene that is inferred to have been present in an ancestral genome, specifically a gene in the last common ancestor of two or more extant species. Thus, given a comprehensive set of gene trees (covering essentially all the genes in a set of extant species), we can infer the set of protein-coding genes that were present in the genome of each common ancestor among these extant species.

The Ancestral Genomes resource provides access to reconstructed genomes for the set of 78 last common ancestors, among a set of 112 different extant species, from bacteria to archaea to eukaryotes. The reconstructions are based on over 15,000 gene trees, covering over 1 million protein-coding genes. Resources currently exist for orthologs (see (5) for examples) and for phylogenetic trees (e.g. PANTHER (6), PhylomeDB (7), and TreeFam (8). However, unlike any other resource of which we are aware, Ancestral Genomes includes not only genes from extant genomes, but represents ancestral genes and genomes explicitly: each ancestral gene is given a stable identifier and reconstructed protein sequence, and is linked to a specific ancestral organism (cross-referenced where possible to multiple resources including NCBI Taxonomy). Here, we describe the overall reconstruction process, and the tools available at http://ancestralgenomes.org for exploring those reconstructions.

## ANCESTRAL GENOME RECONSTRUCTION PROCESS

Ancestral Genomes leverages the extensive development of the PANTHER resource for the past 20 years (6,9). As previously described (10), complete sets of protein coding genes are obtained from the UniProt resource (11), for 112 completely sequenced genomes. These genomes span the tree of life, and include 35 bacteria, 8 archaea, and 69 eukaryotes (covering 9 plants, 37 animals, 16 fungi and 9 protists). Genes are clustered into families based on sequence similarity and human curation, with extensive ongoing feedback from the broader community. This iterative process results in improvements with each successive release. The current version of PANTHER (version 13.1) comprises 15 524 families. A multiple sequence alignment is created for each family. After this stage, however, the information in Ancestral Genomes diverges from that in PANTHER.

The gene trees in AncestralGenomes are reconstructed using a modified version of the GIGA algorithm (12) that is used in PANTHER. The modification adds explicit inferences of gene deletion events in evolutionary history. These inferences are made using the standard evolutionary reconstruction criterion of maximum parsimony: whenever one or more genes are missing from a family tree (i.e. they are expected on the basis of the species tree) the algorithm assumes the smallest number of deletion events needed to explain the missing genes. For example, if a gene tree contains one rat gene, one mouse gene and one gorilla gene, but no gene in either chimpanzee or human, the most parsimonious scenario is that the gene was lost prior to the human-chimp common ancestor, rather than having been present in the human-chimp common ancestor, and subsequently lost independently twice (once in the chimpanzee lineage and once in the human lineage). As a result of this additional processing, the gene trees in Ancestral Genomes also contain an explicit tree node for every inferred ancestral gene, even if it is not a last common ancestor of extant genes due to gene deletions.

Ancestral Genomes maintains stable identifiers for all ancestral genes inferred to be present in each of the last common ancestors of the 112 extant genomes. The PANTHER identifiers for nodes in the tree (identifiers starting with PTN) are used whenever possible, to enable users to access information already available in PANTHER. However, Ancestral Genomes also includes additional stable identifiers for the explicit ancestral genes that are absent from PANTHER due to gene deletion.

Ancestral Genomes provides a reconstruction of the protein sequence encoded by each ancestral gene. Currently, sequences are reconstructed using a local maximum parsimony method. This method works from the leaves toward the root of the tree. We have implemented a simple voting procedure:

- If a plurality of an ancestral node's descendants have the same amino acid at a given site, the ancestor is inferred to conserve that same amino acid.
- If there is a tie vote among descendants, then the nearest outgroup sequence(s) are added to the votes.
- If there is still no plurality after considering the outgroups, then an unknown amino acid symbol (‘X’) is inferred at that site.

One advantage of this simple approach is that insertions and deletions can be treated the same way, and unlike other sequence reconstruction approaches such as maximum likelihood under an evolutionary model (13), we can also infer the occurrence of insertions and deletions of amino acids during evolution. However, we recognize that for the actual amino acid inferences, a maximum likelihood approach would be much better, and we are currently working on integrating the maximum likelihood marginal state reconstructions from Zhang (13,14).

### Interpretation of an ancestral genome

For most of the Ancestral Genomes we have reconstructed, specifically the eukaryotic Ancestral Genomes that are not too ancient (<1000 million years ago, mya), the interpretation is straightforward: these genes were likely present in most individuals of an ancestral species, defined as a population of interbreeding individuals. However, the interpretation is less clear for prokaryotes, and for Ancestral Genomes where the species tree of life is not fully resolved into bifurcating speciation events (e.g. the last eukaryotic common ancestor, that has four distinct lineages descending from it, whose branching order is not yet established). While horizontal gene transfer appears to be relatively rare among eukaryotes (15), it is common among prokaryotes (16). When horizontal transfer is relatively recent, and between species that are distantly related overall, the event can be identified from the gene trees. However, when the transfer is more ancient, or occurred between ancestral species that are not from highly divergent lineages in the species tree, these events will go undetected. As a result, ancestral prokaryotic genomes are likely to represent what Puigbo *et al.* (17) have referred to as a ‘supergenome’ of individuals from different species that could exchange genes via horizontal transfer, so no one organism carried the entire set of genes in the ancestral genome. Even in the absence of horizontal transfer, an ancestral genome will also have a complex interpretation whenever the species tree is multifurcated (non-binary), or speciation is so rapid that incomplete lineage sorting is common. In these cases as well, an ancestral genome is likely to contain more genes than were present in the genome of any single individual. Notable multifurcations in the species tree occur at eubacteria, eukaryota, and eumetazoa. The most extreme example in our reconstruction is the eubacterial ancestral supergenome, which is likely to be affected by both multifurcation, and undetected horizontal transfer following divergence from a common ancestor. We infer ∼6200 genes for the eubacterial supergenome, while the descendant ancestral supergenomes contain only ∼1000 to 5800 genes. This trend has been found in other reconstructions, such as that of Puigbo *et al.* (17), who concluded that high rates of gene loss are a valid interpretation, and in fact the prevailing mode of bacterial evolution.

## COMPARING RECONSTRUCTIONS TO PREVIOUS STUDIES

To assess our reconstructions, we compared them to several published studies from independent groups. Unlike our reconstructions, none of these studies covers the entire tree of life, but they focus on different branches, which together provide a good sampling of the species tree in Ancestral Genomes. Overall, our reconstructions are consistent with the previous findings, including those of Puigbo *et al.* (17) for eubacteria described above.

In the eukaryotes, a number of studies have been carried out that involve gene family evolutionary reconstructions at large scale. These previous studies involve thousands of gene families but are not as comprehensive and complete as the reconstructions in PANTHER, so the most meaningful way to compare their results is to calculate the ratio between a given event inferred in PANTHER, to that in another study. Assuming a study samples randomly from the larger set of families reconstructed by PANTHER, the ratios calculated for different events from the same study should be similar. Zhou *et al.* (18) estimated the history of eukaryotic gene evolution using genes from three plants species, three animals and two fungi. They clustered the genes into families, and found 2600 that included genes from at least two kingdoms. Of these, they found that between 10–20% had good support for gene duplication occurring on the branch prior to the last eukaryotic common ancestor (LECA), depending on the bootstrap or likelihood ratio test threshold. In our reconstruction (PANTHER version 9), we find that of 7178 PANTHER families with the same phylogenetic distribution, 1781 (18%) contain duplications occur before LECA, which is within the range of the reconstructions from Zhou *et al.* Blomme *et al.* (19) reconstructed the history of gene duplication in a large sample of gene families constructed among seven vertebrate genomes. For internal branches of the species tree, we find PANTHER version 9 estimates of duplication events to be in a consistent ratio of about 2 with those of Blomme *et al.*: 4775:2972, about 1.6, for the branch prior to the last common ancestor (LCA) of vertebrates; 1099:545, about 2.0, for the branch prior to the LCA of zebrafish and pufferfish. For terminal branches, we find a substantially larger ratio (1613:363, about 4.4, for the pufferfish branch; 8621:1269, about 6.8, for the zebrafish branch; 3127:217, about 14.4, for the frog branch). We note that terminal branches, compared to internal branches, are much more dependent on genome assembly quality, as low quality can result in apparent recent duplications and deletions. Wyder *et al.* (20) estimated the extent of gene loss during insect evolution, for a sampling of gene families. Again, the ratios comparing different events from this study to our reconstruction method are roughly similar, at roughly 2: 1093:583, about 1.9, for the branch from the LCA of bees and flies to the LCA of flies; 580:306, about 1.9, for the branch prior to the LCA of mosquitoes; 1117:506, about 2.2, for the fruit fly branch). Our estimates of losses along other terminal branches are somewhat higher (666:206, about 3.2, for the yellow fever mosquito branch; 1641:423, about 3.9, for the malaria mosquito branch; 3700:582, about 6.4, for the honeybee branch) but the relationships hold (e.g. more losses in the yellow fever mosquito compared to the malaria mosquito).

## NAVIGATING ANCESTRAL GENOMES

**Figure 1.**
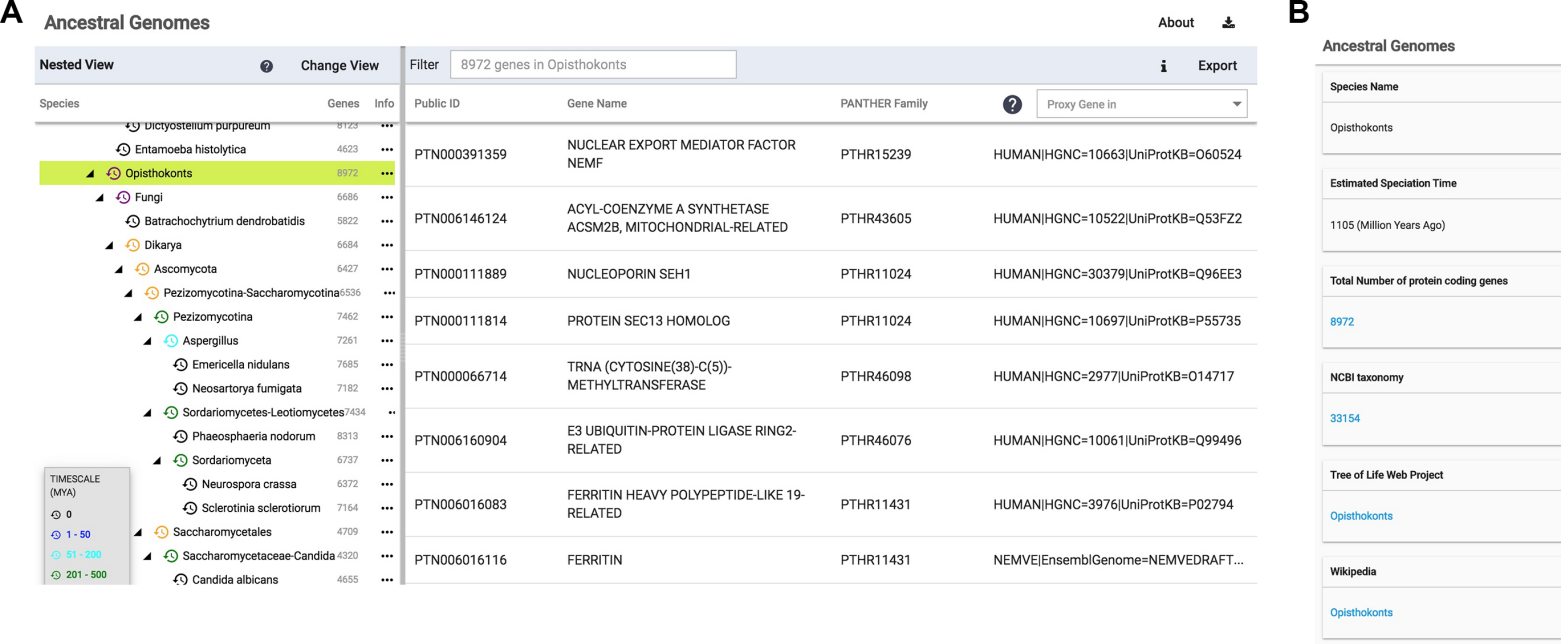
Browsing and selecting an ancestral genome. (**A**) Users can browse the available genomes on the left panel. Clicking on a genome (e.g. Opisthokonts) brings up the list of genes in the right panel. (**B**) Clicking on the ‘…’ (info) button in the left panel in (A) brings up more information about that ancestral organism, including links to information in other resources, and the estimated age of that ancestor (in millions of years ago) as reported in the TimeTree (30) resource.

**Figure 2.**
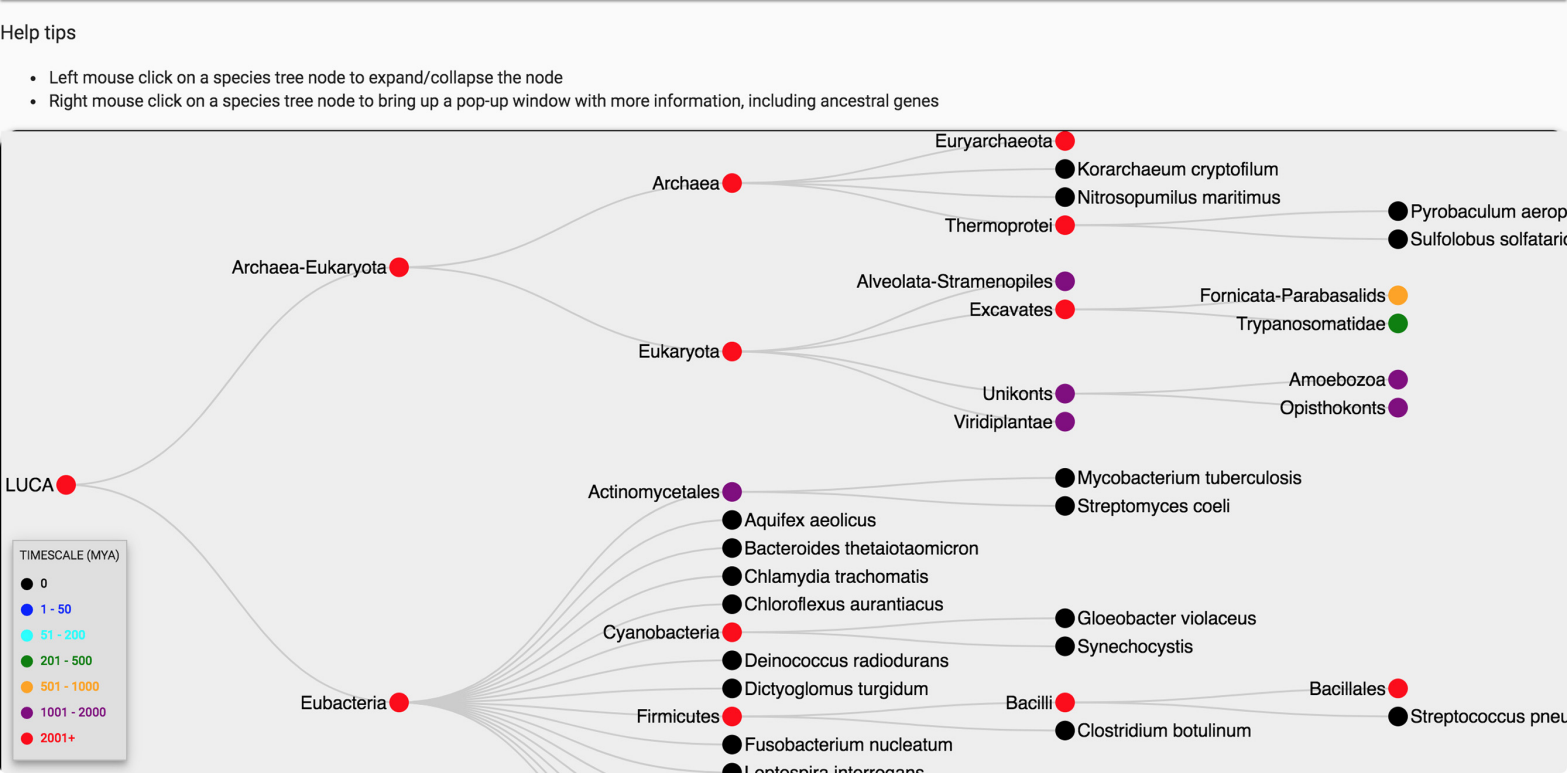
Species Tree Browser. We also provide a species tree view of the genomes in Ancestral Genomes. This view is much less compact than the default nested hierarchical view, but it is a more standard view in phylogenetics. Right-clicking on a node will bring up the ancestral genome overview page (Figure 1B) for that genome, from which users can bring up the list of genes inferred for that genome.

The primary navigation method uses the species tree to select a common ancestral genome. For instance, clicking on ‘Opisthokonts’ will select the genome for the most recent common ancestor of all animals and fungi (Figure 1A), and retrieve a list of genes reconstructed for that ancestor. Users can then search/filter for specific genes by typing in the search field above the list of genes, download the list of genes and associated information, or get more information about that ancestor (Figure 1B). We chose to use a hierarchical, nested view of the species tree by default, rather than the phylogenetic tree view that is more traditional in phylogenetics. The reason for this choice is that the hierarchical view takes much less horizontal screen real estate, and allows users to simultaneously view the list of ancestral genes for the selected genome, and rapidly change their view to a different ancestral genome. The hierarchical view will be familiar to users, as it is used in computer interfaces to navigate file folder hierarchies. However, we also provide a phylogenetic Species Tree View (Figure 2) for navigating and selecting an ancestral genome. In both the Nested View and the Tree View, we use color coding to help orient the user as to the age of each ancestral genome, with the oldest genomes (>2000 mya) in red, and the newest (<50 mya) in blue. Extant genomes are in black.

## ANCESTRAL GENE INFORMATION

**Figure 3.**
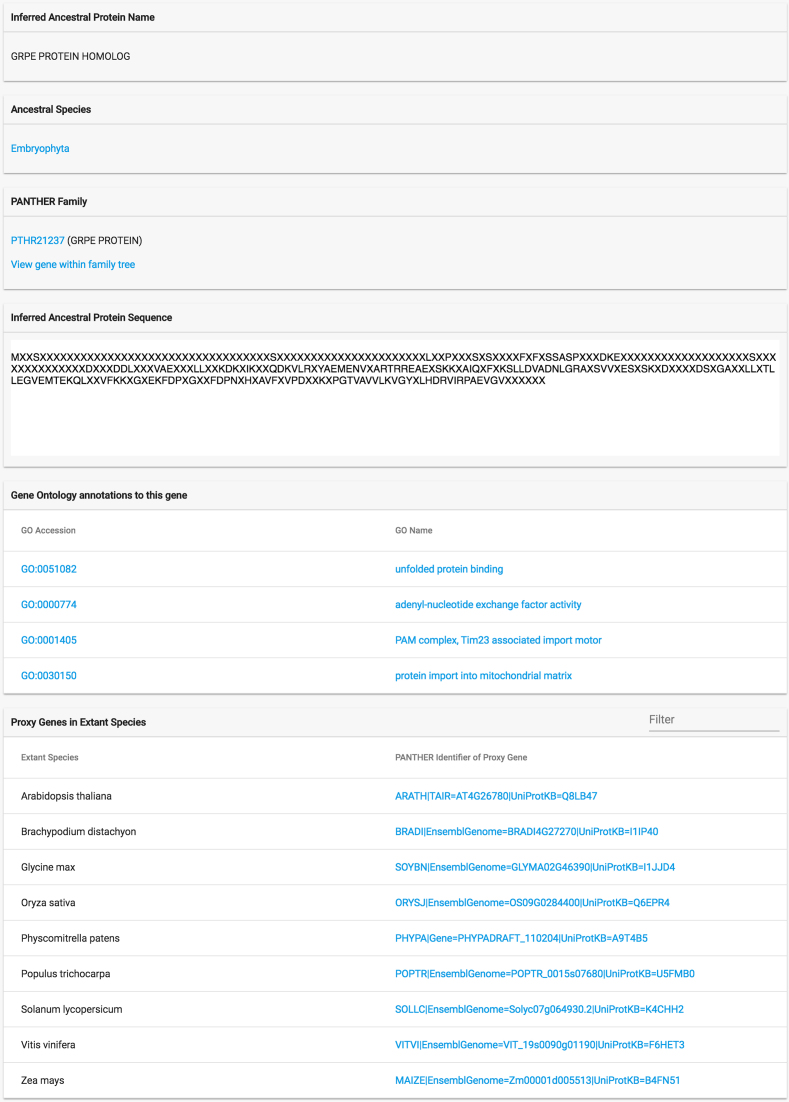
Ancestral gene information page. This page has information about the ancestral gene, including stable identifier, protein name, reconstructed sequence, inferred GO functions and proxy genes.

Selecting an ancestral gene will bring up the gene page for that gene, that contains information about the gene, including the stable identifier and reconstructed sequence as described above (Figure 3). This page also shows gene function information (Gene Ontology terms) that has been inferred for each ancestral gene, by expert curators in the Gene Ontology Phylogenetic Annotation project (21). This project uses the phylogenetic tree, as well as experimentally supported GO annotations, to infer ancestral gain and loss of function. In Ancestral Genomes, we use this information to propagate GO terms from more ancient, to more recent ancestral genes in the tree. In addition, to facilitate browsing and analysis, we also assign a protein name to each ancestral gene whenever possible. Protein names are selected based on an extension of the subfamily naming convention used in PANTHER, as described in Mi *et al.* (10). In brief, whenever a new gene was created by gene duplication (and is present in at least two different genomes, as confirmation of its presence), one of the duplicates is named for its descendant in a well-studied, extant genome. This name is then inherited by its descendants in other extinct genomes, unless another duplication occurs that requires the naming rule to be re-applied.

For each ancestral gene, we also provide a list of its proxy genes in extant genomes. From a practical standpoint, proxy genes provide a way to analyze ancestral genes. Because the ancestral genes themselves are extinct, they have not been studied in the lab. But their descendants in extant genomes may have been studied, particularly if they are from a well-studied ‘model organism’ (22) such as the bacterium *E. coli*, yeast (budding or fission), the plant *A. thaliana*, fruit fly, nematode worm *C. elegans*, mouse, rat or zebrafish. More formally, proxy genes are simply an extension of the well-known comparative biology concept of orthologs, which are widely used to infer properties of an uncharacterized gene in one organism, from a related, experimentally characterized gene in another organism (23). Orthology is a relationship between two extant genes, via the gene that was their last common ancestor. Fitch defined orthologs as two genes in different extant species that can be traced to the same gene in the common ancestor genome of those organisms (24). Proxy genes describe the relationship between an ancestral gene and its extant descendants. We define a proxy gene as the most closely related descendant of that ancestral gene in an extant organism. One could in principle define ‘closest’ in different ways, but for convenience we define it in terms of protein sequence divergence, as this quantity is easily calculated from our trees as total branch length in the path from an ancestral gene to an extant (leaf) descendant.

## COMPARING AN EXTANT GENOME WITH ITS ANCESTRAL GENOMES

**Figure 4.**
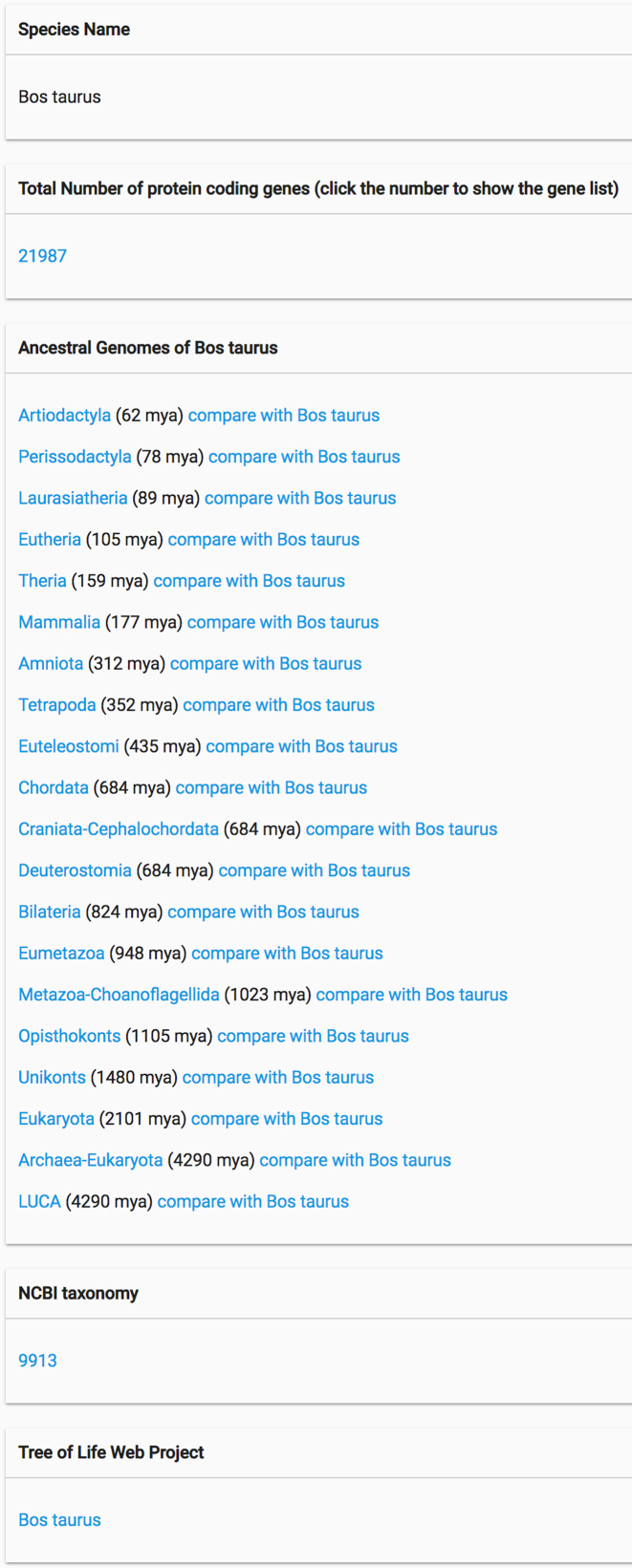
Extant species information page. This page has information about the extant species, including species name, total number of protein coding genes, its ancestral species and their speciation times. Each ancestral species is followed by a link to the genome comparison page.

**Figure 5.**
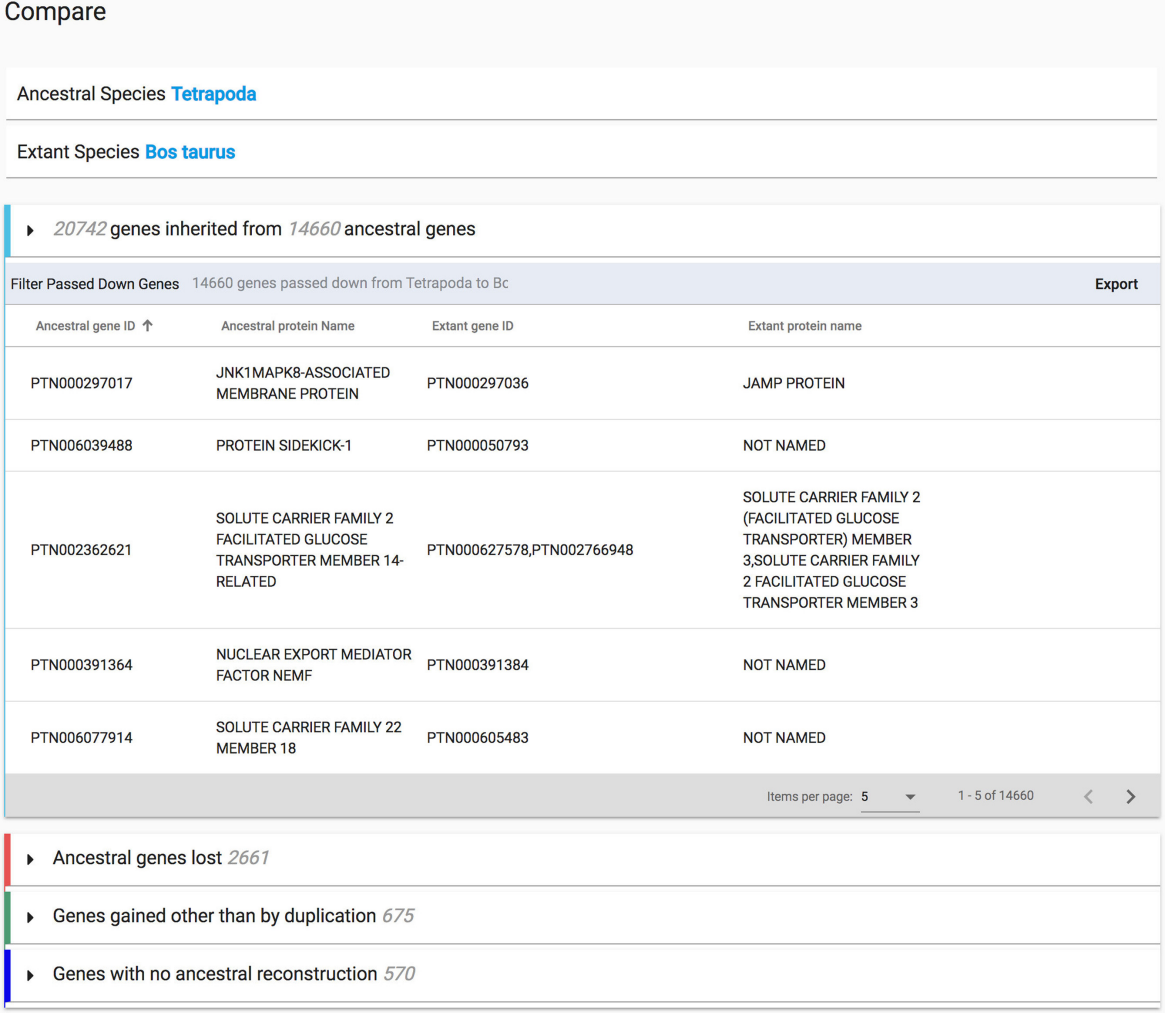
Extant and Ancestral Genomes comparison page. There are four collapsible sections on the page, each with a header displaying summarizing information about the changes in gene content from the ancestral genome to the extant genome. Each section can be expanded to list the genes in that category.

From an extant genome information page, users can view the available ancestral reconstructions in that lineage, leading back to the last universal common ancestor (Figure 4). A link is available to a web page that compares the extant genome to the selected ancestral genome. The comparison page has four separate sections (Figure 5). The first section displays the genes that were inherited in the extant genome from the selected ancestral genome. For each ancestral gene, the table lists the extant gene(s) that can be traced back to that ancestral gene. Note that an ancestral gene can have more than one descendant in the extant genome, if one or more gene duplications have occurred during the time between the ancestor and its descendant. The second section displays the genes in the ancestral genome that were lost during the time between the ancestor and its descendant. The third section displays the genes gained by horizontal transfer or potential ‘*de novo*’ gene evolution: genes that were gained in the extant genome relative to the ancestor, but could not be traced to a reconstructed gene in that ancestor. Note that it is possible that some potential *de novo* gene gains can in fact be traced to an ancestral gene, but for various biological (e.g. high degree of sequence divergence) or technical (e.g. how PANTHER has divided up genes into families) reasons, were not able to be traced. Finally, we include a fourth section that lists the extant genes whose histories have not yet been reconstructed. These are generally genes with few homologs among the >100 species in the PANTHER trees, and therefore represent genes that can only be traced back to relatively recent ancestral genomes.

## GENE TREE VIEWER WITH EXPLICIT REPRESENTATION OF GENE LOSS EVENTS

**Figure 6.**
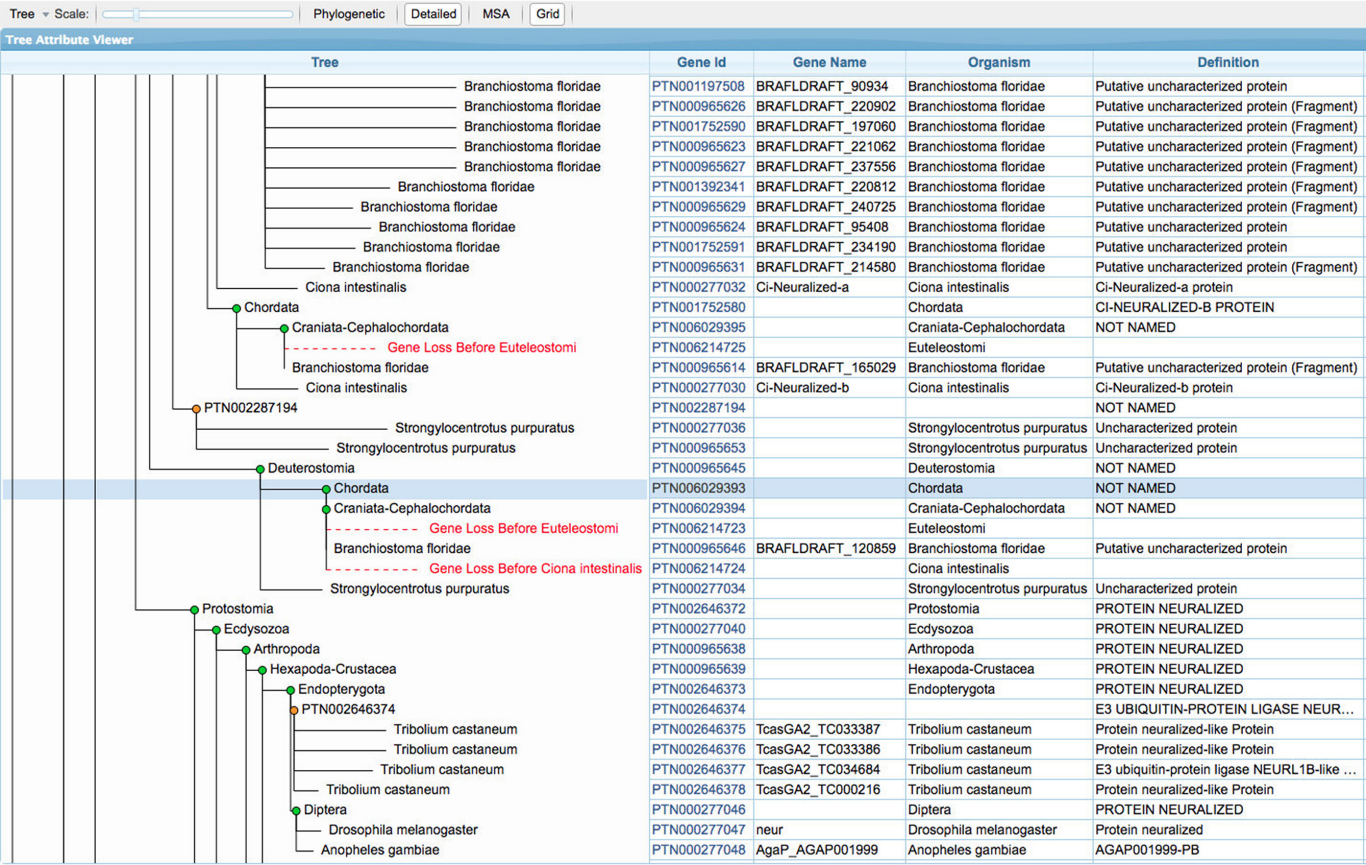
Gene Tree Viewer. Gene loss is shown explicitly in red, with text indicating the ancestral species prior to which the gene was lost.

The gene trees in Ancestral Genomes include explicit representations of inferred gene loss events, a feature that, to our knowledge, is not available in any other resource. At http://ancestralgenomes.org, we provide a gene tree viewer that enables visualization of these gene loss events (Figure 6). Gene losses appear as branches in the tree along which a gene was lost, and are labeled with the first ancestral species that is inferred to have been missing that gene. Because of this visualization convention, the gene trees in Ancestral Genomes also contain an explicit tree node for every inferred ancestral gene, even if it is not a last common ancestor of extant genes due to gene deletions. Note that the Gene Tree Viewer at Ancestral Genomes includes features that enable users to explore protein sequence evolution throughout the tree. The gene trees can be accessed from the ancestral gene information page (Figure 3), and will highlight the selected gene. The Gene Tree Viewer uses a nested hierarchical view ('Detailed') by default, which allows the display of table view with information about each ancestral (and extant) gene. Users can toggle back to the standard ‘Phylogenetic’ view with one click. Users can select the multiple sequence alignment viewer to see the reconstructed amino acid sequences of ancestral genes, and explore how those sequences have evolved.

## CONCLUSIONS

We have created a new resource for exploring reconstructed Ancestral Genomes across the tree of life. For the first time, the scientific community can easily access a current ‘best estimate’ as to the number of protein coding genes present in Ancestral Genomes across the tree of life, and can explore the reconstructed genes in terms of their extant descendants, and even their reconstructed protein sequences. Users should understand some of the caveats of these reconstructions. Sampling of genomes across the tree of life can affect the accuracy of reconstructions: branches that are under-sampled, such as bacteria and archaea, will be less accurate. Because reconstruction methods rely on detection of homology at the protein sequence level, if homologs diverge too much in sequence, their relationships will not be detected. As a result, we are likely to underestimate the gene content of some ancestral genomes, particularly those that are more ancient.

We recognize that ancestral genome reconstruction is an ongoing and active area of research, and we expect that the accuracy of our reconstructions will continue to improve over time. The reconstructions in the Ancestral Genomes resource will be updated yearly, in synchronization with the PANTHER database. Improvements in the sets of genes available from the UniProt Reference Proteomes data source will result in improved reconstructions. Improvements are also expected in the gene trees due to ongoing feedback from manual curation in the GO Phylogenetic Annotation project (21), the sharing of data between PANTHER and Ensembl Compara (25), and orthology benchmarking in the Quest for Orthologs Consortium (5). Although we have used the PANTHER pipeline for our initial reconstructions, we recognize that other methods, such as using different methods for multiple alignment (26) or construction of reconciled gene trees under a deletion-transfer-loss model (27,28) may result in differences in the inferred ancestral gene content, as well as in the inferred ancestral gene sequences (29). We encourage community feedback on our reconstructions, and will aim to incorporate feedback in future releases.
